# The Diagnostic Ability of Follow-Up Imaging Biomarkers after Treatment of Glioblastoma in the Temozolomide Era: Implications from Proton MR Spectroscopy and Apparent Diffusion Coefficient Mapping

**DOI:** 10.1155/2015/641023

**Published:** 2015-09-13

**Authors:** Martin Bulik, Tomas Kazda, Pavel Slampa, Radim Jancalek

**Affiliations:** ^1^Department of Diagnostic Imaging, Faculty of Medicine, Masaryk University, 625 00 Brno, Czech Republic; ^2^Department of Diagnostic Imaging, St. Anne's University Hospital Brno, 656 91 Brno, Czech Republic; ^3^International Clinical Research Center, St. Anne's University Hospital Brno, 656 91 Brno, Czech Republic; ^4^Department of Radiation Oncology, Faculty of Medicine, Masaryk University, 625 00 Brno, Czech Republic; ^5^Department of Radiation Oncology, Masaryk Memorial Cancer Institute, 656 53 Brno, Czech Republic; ^6^Department of Neurosurgery, St. Anne's University Hospital Brno, Faculty of Medicine, Masaryk University, 625 00 Brno, Czech Republic; ^7^Department of Neurosurgery, St. Anne's University Hospital Brno, 656 91 Brno, Czech Republic

## Abstract

*Objective*. To prospectively determine institutional cut-off values of apparent diffusion coefficients (ADCs) and concentration of tissue metabolites measured by MR spectroscopy (MRS) for early differentiation between glioblastoma (GBM) relapse and treatment-related changes after standard treatment. *Materials and Methods*. Twenty-four GBM patients who received gross total resection and standard adjuvant therapy underwent MRI examination focusing on the enhancing region suspected of tumor recurrence. ADC maps, concentrations of *N*-acetylaspartate, choline, creatine, lipids, and lactate, and metabolite ratios were determined. Final diagnosis as determined by biopsy or follow-up imaging was correlated to the results of advanced MRI findings. *Results*. Eighteen (75%) and 6 (25%) patients developed tumor recurrence and pseudoprogression, respectively. Mean time to radiographic progression from the end of chemoradiotherapy was 5.8 ± 5.6 months. Significant differences in ADC and MRS data were observed between those with progression and pseudoprogression. Recurrence was characterized by *N*-acetylaspartate ≤ 1.5 mM, choline/*N*-acetylaspartate ≥ 1.4 (sensitivity 100%, specificity 91.7%), *N*-acetylaspartate/creatine ≤ 0.7, and ADC ≤ 1300 × 10^−6^ mm^2^/s (sensitivity 100%, specificity 100%). *Conclusion*. Institutional validation of cut-off values obtained from advanced MRI methods is warranted not only for diagnosis of GBM recurrence, but also as enrollment criteria in salvage clinical trials and for reporting of outcomes of initial treatment.

## 1. Introduction

High-grade gliomas (HGG) are the most common and the most serious of primary brain tumors. Despite significant improvements in patient outcomes associated with the introduction of temozolomide (TMZ) into treatment protocols, prognosis remains dismal. The median progression-free survival of glioblastoma (GBM), the most common and lethal HGG, is still only 6.9 months [1]. Unfortunately, with conventional MRI, recurrences often have similar radiologic characteristics as therapy-related changes such as pseudoprogression (PsP) or radionecrosis, and its mutual differentiation remains challenging [2].

Routinely available structural MRI utilizing T2- and gadolinium-enhanced T1-weighted sequences has insufficient sensitivity and specificity for differentiation between recurrence and radionecrosis or PsP, due to their similar imaging patterns characterized by contrast-enhancing lesion(s) surrounded by edema [3, 4]. PsP can develop after radiotherapy alone but more frequently is present after concomitant radiotherapy and TMZ with occurrence in up to 30% of patients, especially those with O(6)-methylguanine-DNA methyltransferase (MGMT) promoter methylation [5, 6]. Even higher incidence of unclear early radiographic progression at the first postradiotherapy imaging was reported [7]. Nevertheless, in the most recent and robust analysis performed by researchers from Heidelberg, PsP incidence was indicative of prolonged overall survival, despite quite low overall (11.4% of 79 patients) [8]. Thus, the valid and accurate differentiation of follow-up lesions becomes increasingly important for the proper indication of subsequent management, especially in countries with regulatory approval of bevacizumab for salvage treatment [9].

Modern multiparametric MRI techniques such as diffusion-weighted imaging (DWI) with apparent diffusion coefficient (ADC) mapping, dynamic susceptibility-weighted contrast-enhanced (DSC) perfusion imaging, and MR spectroscopy (MRS) allow a much deeper and still noninvasive insight into interpretation of brain lesions, resulting in greater specificity of diagnostic imaging, especially when in combination with amino acid PET imaging [10–14].

However, in routine practice, availability of advanced MRI as well as PET methods is limited with exception of DWI/ADC and MRS. DWI reflects changes in water diffusion as a result of changed tissue microarchitecture due to tumor infiltration and can be quantitatively assessed with the ADC. MRS enables noninvasive examination of the spatial distribution of multiple metabolite concentrations in normal and pathological tissues. The goals of the present prospective study are to verify whether combination of ADC values and concentrations of tissue metabolites measured by proton MRS enable early differentiation between GBM relapse and treatment-related changes in the era of routinely used TMZ and to set institutional cut-off values for increasing accurate diagnosis.

## 2. Materials and Methods

### 2.1. Patient Selection and Treatment

Consecutive series of patients with GBM underwent standard treatment consisting of maximal safe resection at the Department of Neurosurgery at St. Anne's University Hospital Brno followed by adjuvant concurrent chemotherapy and radiation therapy (RT). Only patients with MRI-proven gross total resection were eligible. TMZ was administered daily during RT and 5 days every 4 weeks for six cycles as adjuvant treatment. RT was delivered by linear accelerator to the standard dose of 60 Gy in 30 fractions to the clinical target volume defined as the resection cavity with a margin of 1-2 cm. The T2/FLAIR signal abnormality received 40–50 Gy while meeting dose constraints for adjacent organs at risk. Patients underwent structural MRI 6 weeks after the end of RT and then every 3 months thereafter. After radiographic progression was determined with structural MRI, patients became eligible for receiving MRS and DWI. At the treating physician's discretion, biopsy/resection or repeated structural MRI was performed in the final determination of progression. The protocol for this prospective study was approved by St. Anne's University Hospital Brno Institutional Review Board and informed consent was signed by all enrolled patients.

### 2.2. Advanced MRI

Advanced MRI and proton MR spectroscopy examinations were performed using a 3.0T clinical MR scanner (GE Medical Systems Discovery MR750). Due to the signal heterogeneity and irregular shape of observed MRI lesions, 2D proton MR spectroscopy maps covering the gadolinium-enhanced regions on MRI were performed by means of chemical shift imaging (CSI) technique in two orthogonal planes respecting long axis of the lesion and proximity to structures increasing noise in MR spectra (e.g., bone tissue). All voxels covering the region marked by experienced neuroradiologist as suspected of GBM relapse or PsP were analyzed and the representative ones with the lowest signal-to-noise ratio on each MR spectroscopy map were chosen for further analysis. This procedure led to two spatially independent concentrations of measured metabolites in each patient and resulted in a total of 48 original values for each metabolite in the cohort of 24 patients.

The following parameters were used for proton MR spectroscopy: a point-resolved spectroscopy sequence (PRESS), TR/TE 1800/144 ms, 16-cm FOV, 15-mm slice thickness, and voxel size 10 × 10 × 15 mm. The volume of interest (VOI) encompassed the contrast-enhancing region in contrast-enhanced axial T1-weighted images. Automatic prescanning was performed prior to each spectroscopic scan to ensure adequate water suppression.

MR spectroscopy data were evaluated using LCModel version 6.3 [15] and the concentration of each metabolite was measured. The LCModel data were further postprocessed by jSIPRO 1.0_beta [16]. Metabolite peaks were identified for* N*-acetylaspartate,* N*-acetylaspartylglutamate (tNAA), choline-containing compounds (tCho), (phospho-)creatine (tCr), lipid-containing compounds at 1.3–0.9 ppm (Lip), and lactate (Lac). Metabolite ratios were calculated manually. A routine water unsuppressed spectrum obtained at each examination was used to evaluate the spectrum quality.

The DWI scans were obtained by using an axial echo-planar SE sequence (TR/TE 6000/100 ms), 5-mm slice thickness, diffusion gradient encoding in three orthogonal directions, *b* = 0 and 1000 mm^2^/s, and 240-mm FOV. Postprocessing of DWI data with calculation of ADC maps was performed by using OsiriX software version 6.0.2 64-bit (Pixmeo SARL, Switzerland) with ADC Map Calculation plugin version 1.9 (Stanford University). Regions of interest (ROIs) were drawn manually onto the ADC maps and corresponded to the MRS voxels covering areas with contrast enhancement on T1-weighted images. The mean ADC value (ADCmean) in the voxel corresponding with the measured MRS voxel was calculated automatically by OsiriX software.

### 2.3. Data Analysis

The metabolite concentrations, their ratios, and ADCmean values were further evaluated using statistical software STATISTICA 12 (StatSoft, Inc.) and expressed as medians. Fisher's exact test for categorical data and Mann-Whitney *U* test for continuous variables were used for estimation of significance of measured differences. ROC analysis was used for definition of the optimal diagnostic cut-offs and description of their sensitivity and specificity for the final diagnosis. The area under the ROC curve (AUC) expressed a measure of how well a parameter can distinguish between the two diagnostic groups (GBM relapse and PsP). Probability value *p* < 0.05 was considered significant in all tests.

## 3. Results

### 3.1. Patient Characteristics

**Figure 1:**
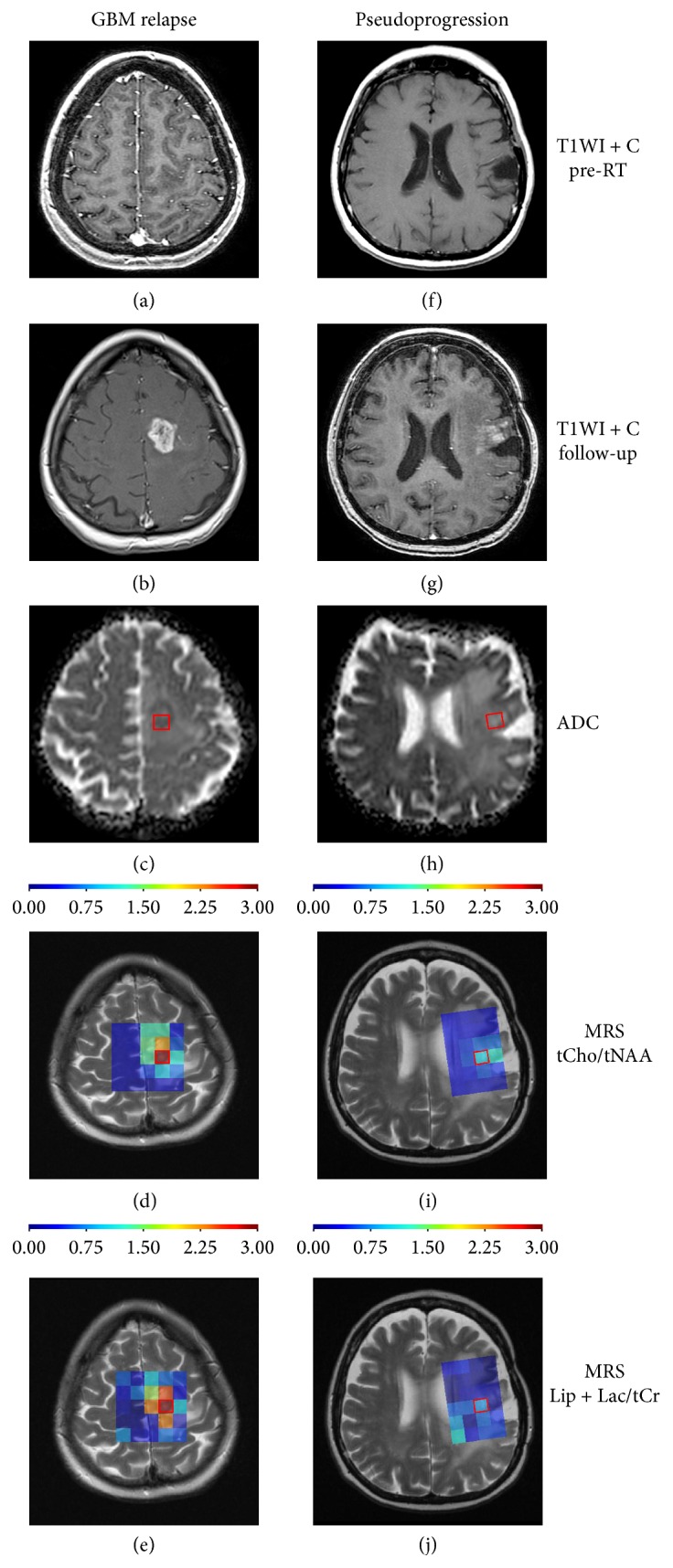
Representative MRI examples of glioblastoma relapse ((a)–(e)) and pseudoprogression ((f)–(j)): (a) + (f) show T1WI with gadolinium after surgical resection before radiotherapy, (b) + (g) show follow-up T1WI with gadolinium after 3 months from radiotherapy, (c) + (h) show ADC maps with marked VOI (ADCmean values for VOI: 848 × 10^−6 ^mm^2^/s in GBM relapse and 1355 × 10^−6 ^mm^2^/s in PsP), (d) + (i) show proton MR spectroscopy maps focused on tCho/tNAA ratio with marked VOI (peak values: 2.98 in GBM relapse and 1.33 in PsP), and (e) + (j) show proton MR spectroscopy maps focused on Lip + Lac/tCr ratio with marked VOI (peak values: 2.93 in GBM relapse and 0.83 in PsP).

Twenty-four patients (mean age 52 years) were enrolled between May 2013 and August 2014. Their characteristics are summarized in Table 1. Sixteen (67%) and 8 (33%) patients had their final diagnosis made by biopsy/resection and by imaging findings on subsequent structural MRI, respectively. Eighteen (75%) patients developed tumor recurrence, 6 (25%) developed PsP, and none developed radionecrosis. Representative imaging data of patients are shown in Figure 1. With 13.8 months of median overall survival, the mean time to radiographic progression from the end of chemoradiotherapy was 5.8 ± 5.6 months. Zero and 9 (37%) patients developed radiographic progression during the first 6 weeks and during the first 3 months after the end of RT, respectively.

### 3.2. ADC and MRS

Relapse of GBM was characterized by a significantly lower concentration of tNAA as compared to PsP (*p* < 0.001; Table 2), with a cut-off of 1.5 mM (sensitivity 75%, specificity 100%). While only 25% of the patients with a GBM relapse had a concentration of tNAA > 1.5 mM, all of the patients with PsP had [tNAA] > 1.5 mM (Table 3). GBM relapse was also characterized by a higher concentration of Lip + Lac compared to PsP with a cut-off 4.8 mM (sensitivity 100.0, specificity 66.7) (*p* = 0.004; Table 2). Although 33.3% of patients with PsP had a Lip + Lac concentration ≥ 4.8 mM, all patients with GBM relapse had a Lip + Lac concentration ≥ 4.8 mM (Table 3). Concentrations of tCho and tCr did not reach statistical significance between the two groups of patients.

The findings from the individual metabolites were also seen in their ratios. The tCho/tNAA, tNAA/tCr, and Lip + Lac/tCr ratios showed significant differences between GBM relapse and PsP (*p* < 0.001, *p* < 0.001, and *p* = 0.004, resp.) (Table 2). GBM relapse was characterized by a lower tNAA/tCr ratio with a cut-off of 0.7 (sensitivity 94.4%, specificity 91.7%) and higher Lip + Lac/tCr ratio with a cut-off of 1.9 (sensitivity 91.7%, specificity 75.0%; Table 2). Moreover, the GBM relapse group had higher tCho/tNAA ratio values (cut-off 1.4; sensitivity 100.0%, specificity 91.7%; Table 2). Whereas a tCho/tNAA ratio < 1.4 was not specific for PsP, all patients with GBM relapse had a tCho/tNAA ratio ≥ 1.4 (Table 3). The tCho/tCr ratio did not reach statistical significance between both groups of patients.

The calculated ADCmean value was significantly lower in the GBM relapse group than in the PsP group (*p* < 0.001) with a cut-off of 1300 × 10^−6 ^mm^2^/s (sensitivity 100.0%, specificity 100.0%; Table 2). All patients with GBM relapse had an ADCmean ≤ 1300 × 10^−6 ^mm^2^/s and all patients with PsP had an ADCmean > 1300 × 10^−6 ^mm^2^/s.

## 4. Discussion

Accurate and timely identification of progression is essential for appropriate salvage management for patients with primary brain tumors. Development of response assessment tools is an ongoing process. Currently the most reliable and robust criteria for disease progression are the Response Assessment in Neuro-Oncology (RANO) 2D criteria established in 2010, updated from the earlier established McDonald criteria [17, 18]. In particular, the newly recognized phenomenon of PsP (the transient treatment-related increase of contrast enhancement suggestive of tumor progression) and pseudoresponse (the early and rapid decrease of contrast enhancement without a true tumoricidal effect) are addressed in the RANO criteria. This pseudoresponse is most likely related to the introduction of TMZ and antiangiogenic targeted therapies in treatment protocols [19, 20]. Still, many questions remain for the clear and safe clinical use of TMZ and antiangiogenic targeted therapies. With developments in RT techniques and with the standard administration of TMZ in all GBM patients, radionecrosis has become more infrequent in contrast to the increasing incidence of PsP in therapy-related imaging patterns. Increased incidence of PsP has been proven by several authors especially in tumors with hypermethylation of the O(6)-methylguanine-DNA methyltransferase (MGMT) promoter gene [6] confirming greater activity of the combined treatment in this subset of patients with a favorable prognosis and longer progression and survival times [21]. MGMT is involved in the repair of DNA damage caused by alkylating agents such as TMZ. Methylation of MGMT promoter alters transcription of this gene and inhibits the repair mechanism. According to Kong's results, dynamic susceptibility-weighted contrast-enhanced perfusion MRI can be used for PsP development prediction and for its differentiation from tumor progression in GBM patients. Its value was significantly higher in the patients with an unmethylated MGMT promoter, compared with tumors with a hypermethylated MGMT status [22].

In our series, radionecrosis was not observed in any patients whereas PsP was documented in 25%. However, the RANO definition of PsP, which includes new enhancement within the radiation field within the first 12 weeks after completion of RT, is currently being challenged by Radbruch's observations of considerably lower incidence in PsP compared to previous reports [8]. Furthermore, in 30% of patients PsP developed later than during the first 12 weeks [8]. Taken together, standard follow-up MR imaging of GBM patients has its inherent limitations in identifying PsP with the recommended RANO criteria. The incorporation of advanced MRI techniques into MRI response assessment tools may be warranted for increased sensitivity and specificity in distinguishing between true tumor recurrence and treatment-related changes [10, 23].

Although advanced imaging modalities such as multiparametric MRI and PET have potential for further improvement in evaluation of brain lesions, their limited availability limits their routine use in worldwide clinical practice. For example, the most studied PET tracer for brain tumors, L-[methyl-11C]methionine, has sufficient tumor to normal brain uptake ratio for the recurrence diagnosis, but the short physical half-life of 11C restricts its clinical use to PET facilities that operate a cyclotron for on-site manufacturing of 11C [24]. In contrast, DWI and MRS are becoming a part of standard protocols. These methods enable further brain imaging beyond structural T1 or T2/FLAIR weighted imaging. By measurement of Brownian random motion of water molecules, DWI identifies changes in water diffusivity as a function of surrounding micro architecture such as increases in cell density, a histopathologic characteristic of a tumor recurrence. Decreased diffusivity is reflected in lower ADC values, which are a quantitative parameter of DWI independent of magnetic field strength. In our study, the upper threshold for GBM relapse determined was 1300 × 10^−6 ^mm^2^/s. All patients with PsP had ADCmean values above this cut-off value, yielding 100% sensitivity as well as specificity. This high sensitivity and specificity may be related to the lack of radionecrosis cases in our cohort and its small sample size. It may be assumed that, in PsP cases, the treatment-related tumor vasculature permeability and blood brain barrier instability responsible for temporary contrast enhancement lead to increased intercellular edema (and thus to high ADCs) as compared to radionecrotic cases, where release of products of cell death into the extracellular space may limit water diffusion (and lead to lower ADCs compared to PsP). Thus, it may not be possible to distinguish between recurrence and radionecrosis with 100% specificity and sensitivity utilizing only DWI. The combination of ADC values with MRS focused mainly on tNAA concentration, as a biomarker of neuronal density and viability, may aid in resolving these obscure cases. In our cohort, all PsP patients had tNAA concentration higher than calculated cut-off value, 1.5 mM. However, there are some limitations in the reproducibility and application of absolute metabolite concentrations because of their interpersonal variability [25]. Moreover, a significant regional variability in the absolute metabolite concentrations of different brain regions has to be also taken into consideration [26]. We recommend use of metabolite ratios that have generally lower intrasubject coefficient of variation and thus they can serve as feasible biomarkers for differentiation of PsP and tumor recurrence. Apart from the most common metabolite ratios as tCho/tNAA and tNAA/tCr, which can be correlated with other institutional data (Table 4), we have also documented Lac + Lip/tCr ratio as a new statistically significant parameter (*p* = 0.004) for differentiation between GBM relapse and PsP. GBM relapse was characterized by a higher Lac + Lip/tCr ratio with a cut-off of 1.9 (sensitivity 91.7%, specificity 75.0%; Table 2). This finding indicates lactate and lipid accumulation that is the typical feature of high-grade gliomas documented, in line with our results, by other authors [27].

This small prospective imaging study has two main limitations. One is the lack of standardized MR image acquisition parameters at different institutions which precludes direct comparison with other studies (Table 4). As expected, greater similarity is observed between cross-institutional ADC values than between MRS metabolite concentrations and ratios, which are more sensitive to institutional setup of acquisition parameters. Another limitation is missing biopsy data of suspected lesions in 33% of patients. Imaging of this subgroup of patients with no resolving contrast enhancement may represent a local mixture of PsP patterns and growing recurrent tumor leading to relatively low ADC values, but still MRS characteristics favoring diagnosis of PsP. Unfortunately, this subgroup of patients where biopsy is risky forms the group of patients that would benefit most from the noninvasive nature of advanced MRI. However, care must be taken in the case where different MRI methods point towards different diagnoses. Thus, combination of multiple MRI methods is warranted. Close follow-up with early repeated imaging is recommended for these patients.

The accurate determination of progression is important not only for the individual care of each patient but also for correct enrollment in clinical trials investigating salvage treatment and reporting results of trials investigating initial treatment. While overall survival is generally the most well-established outcome of oncologic clinical trials, time to progression, progression-free survival, and progression-free survival at 6 months are becoming more reasonable endpoints in evaluating brain tumor response [7, 28, 29]. For evaluation of initial treatment (surgery or concurrent chemoradiation), progression is a more accurately representative endpoint compared to overall survival, which may be biased by different salvage treatments. The appropriate and correct determination of progression continues to be essential as well for correct patient enrollment and treatment within salvage treatment clinical trials. Care must be taken in the case of a suspected treatment-related change, which typically results in termination of ongoing effective adjuvant treatment and if misidentified would bias results of the investigated salvage agent. We suggest that institutions involved in clinical research of new agents for patients suffering from brain tumors consider establishing their own institutional validation using advanced MRI methods with institutionally determined cut-off values as in our presented study. Particularly for MRS values, institutionally determined threshold values may be necessary to account for variability between different institutions as summarized in Table 4. Thus, the ratio of concentrations of representative metabolites (e.g., tCho/tNAA) is preferred in comparing the absolute concentration of a metabolite.

In summary, more accessible advanced MRI methods such as diffusion-weighted and spectroscopic imaging may further improve sensitivity and specificity of standard imaging in diagnosing recurrence of brain tumors. ADCmean values ≤ 1300 × 10^−6 ^mm^2^/s and tCho/tNAA ratio ≥ 1.4 are strongly associated with differentiating GBM recurrence from treatment-related changes indicative of PsP. Institutional validation of thresholds for advanced MRI methods is warranted especially for appropriate enrollment into salvage clinical trials and reporting of outcomes of initial treatment.

## Figures and Tables

**Table 1:** Demographic and clinical characteristics: T = temporal, F = frontal, P = parietal, O = occipital, F-P = frontoparietal, 3D-CRT = three-dimensional conformal radiotherapy, and IMRT = intensity-modulated radiotherapy.

Characteristic	*n* = 24
Age at initial diagnosis (years)	
Median	52.5
Range	29–66
Sex (*n*)	
Men	17 (65%)
GBM location (%)	
T/F/P/O/F-P	36/28/21/7/8
Radiotherapy	
Median dose (Gy)	60
Technique 3D-CRT/IMRT (%)	50/50
Cycles of adjuvant TMZ	
Median	6
Range	4–10
Time to graphic progression (months)	
Mean	5.8
SD	5.6
Diagnosis validation	
Biopsy/subsequent imaging (%)	67/33
Final diagnosis	
Tumor recurrence	18 (75%)
Pseudoprogression	6 (25%)

**Table 2:** The cut-offs, sensitivity, and specificity of the metabolite concentrations, their ratios, and ADCmean values in a GBM relapse. AUC, area under the curve for each ROC analysis with appropriate statistical significance (*p*).

	AUC (95% CI)	*p*	Cut-off	Sensitivity	Specificity
Metabolite/MRS			[mM]		

tCho	0.532 (0.325; 0.740)	0.739	≤2.9	69.4	41.7
tNAA	0.970 (0.926; 1.000)	<0.001	≤1.5	75.0	100.0
tCr	0.613 (0.426; 0.801)	0.243	≤2.6	55.6	66.7
Lip + Lac	0.782 (0.574; 0.991)	0.004	≥4.8	100.0	66.7
tCho/tNAA	0.991 (0.970; 1.000)	<0.001	≥1.4	100.0	91.7
tCho/tCr	0.597 (0.388; 0.806)	0.317	≥0.7	83.3	41.7
tNAA/tCr	0.926 (0.786; 1.000)	<0.001	≤0.7	94.4	91.7
Lip + Lac/tCr	0.782 (0.574; 0.990)	0.004	≥1.9	91.7	75.0

ADC/DWI			[10^−6^ mm^2^/s]		

ADCmean	1.000 (1.000; 1.000)	<0.001	≤1300	100.0	100.0

**Table 3:** Comparison of MRS and DWI/ADCmean results between the patients (*n* = 24) with a pseudoprogression and glioblastoma relapse. Two spatially independent values corresponding with two perpendicular planes on MRI were analyzed in each patient (*n* = 48 analyzed samples).

	Pseudoprogression (*N* = 12)	GBM relapse (*N* = 36)	p
tCho [mM]			0.500
>2.9	5 (41.7%)	11 (30.6%)	
≤2.9	7 (58.3%)	25 (69.4%)	
Median (min; max)	**2.88 (0.86; 3.73)**	**2.41 (1.26; 4.40)**	**0.739**
tNAA [mM]			
>1.5	12 (100.0%)	9 (25.0%)	<0.001
≤1.5	0 (0.0%)	27 (75.0%)	
Median (min; max)	**2.88 (1.52; 5.13)**	**1.19 (0.44; 2.22)**	**<0.001**
tCr [mM]			
>2.6	8 (66.7%)	16 (44.4%)	0.318
≤2.6	4 (33.3%)	20 (55.6%)	
Median (min; max)	**2.74 (1.71; 7.53)**	**2.49 (1.46; 5.86)**	**0.243**
Lip + Lac			
<4.8	8 (66.7%)	0 (0.0%)	<0.001
≥4.8	4 (33.3%)	36 (100.0%)	
Median (min; max)	**3.50 (0.31; 26.76)**	**10.77 (5.14; 37.23)**	**0.004**
tCho/tNAA			
<1.4	11 (91.7%)	0 (0.0%)	<0.001
≥1.4	1 (8.3%)	36 (100.0%)	
Median (min; max)	**0.77 (0.38; 1.77)**	**2.00 (1.63; 3.93)**	**<0.001**
tCho/tCr			
<0.7	5 (41.7%)	6 (16.7%)	0.113
≥0.7	7 (58.3%)	30 (83.3%)	
Median (min; max)	**0.82 (0.30; 1.37)**	**0.86 (0.54; 1.64)**	**0.317**
tNAA/tCr			
>0.7	11 (91.7%)	2 (5.6%)	<0.001
≤0.7	1 (8.3%)	34 (94.4%)	
Median (min; max)	**0.99 (0.28; 1.59)**	**0.45 (0.24; 0.72)**	**<0.001**
Lip + Lac/tCr			
<1.9	9 (75.0%)	3 (8.3%)	<0.001
≥1.9	3 (25.0%)	33 (91.7%)	
Median (min; max)	**0.88 (0.08; 12.35)**	**4.43 (1.33; 17.42)**	**0.004**
ADCmean [10^−6^ mm^2^/s]			
>1300	12 (100.0%)	0 (0.0%)	<0.001
≤1300	0 (0.0%)	36 (100.0%)	
Median (min; max)	**1373 (1317; 1463)**	**1160 (1011; 1276)**	**<0.001**

**Table 4:** Comparison of MRS and ADC results with other studies focusing on differentiation of GBM recurrence and treatment-related changes: No. pt. = number of patients, Dg = diagnosis, RI = radiation injury, GR = glioma recurrence, Tu = tumor, and RN = radiation necrosis.

Authors	Primary grade [No. pt.]	MR [T]	Dg	*N*	Cho/Cr	Cho/NAA	NAA/Cr	ADC [10^−3^ m^2^/s]
Hein et al. [30]	III/10	1.5	GR	12				1.18 ± 0.13
	IV/8		RI	6				1.40 ± 0.17

Weybright et al. [31]	II–IV/24	1.5	GR	16	2.52 (1.66–4.26)	3.48 (1.70–6.47)	0.79 (0.47–1.15)	
	Other/5		RI	13	1.57 (0.72–1.76)	1.31 (0.83–1.78)	1.22 (0.94–1.69)	

Zeng et al. [32]	III/36	3.0	Tu	32	2.82 ± 0.65	3.52 ± 0.98	0.84 ± 0.23	1.20 ± 0.08
	IV/19		RI	23	1.61 ± 0.34	1.55 ± 0.54	1.10 ± 0.26	1.39 ± 0.09

Nakajima et al. [33]	II/4	1.5	GR	7	3.17 ± 0.83			
	III/6, IV/8		RN	11	2.25 ± 0.80			

Bobek-Billewicz et al. [34]	III/6	1.5/3.0	GR	5	2.16 (1.67–3.15)	1.9 (0.86–2.36)		1.06 ± 0.18
	IV/2		RI	6	1.34 (1.13–2.37)	2.1 (0.97–2.87)		1.13 ± 0.13

Amin et al. [35]	II/5	1.5	GR	18	2.00 ± 0.20	1.60 ± 0.27		
	III/12, IV/7		RN	6		0.94 ± 0.3		

Present study, 2015	IV/24	3.0	GR	18	0.95 ± 0.27	2.20 ± 0.55	0.45 ± 0.13	1.152 ± 0.064
			RI	6	0.82 ± 0.34	0.86 ± 0.37	1.03 ± 0.38	1.383 ± 0.045
